# Gingival fibroblast suppress the osteogenesis process mediated by bone substitute materials via WNT/β-catenin signaling pathway *in vitro* and *in vivo*

**DOI:** 10.3389/fbioe.2025.1521134

**Published:** 2025-02-10

**Authors:** Guanqi Liu, Jiahui Lin, Xiaoyan Chen, Runheng Liu

**Affiliations:** Hospital of Stomatology, Guanghua School of Stomatology, Sun Yat-sen University and Guangdong Provincial Key Laboratory of Stomatology, Guangzhou, China

**Keywords:** gingival fibroblasts, bone substitute materials, mandible mesenchymal stem cells, osteogenesis, Wnt/β-catenin signaling pathway

## Abstract

**Background:**

The regeneration of bone tissue is a critical challenge in oral and maxillofacial surgery, with the success of such procedures often depending on the ability to promote osteogenesis while managing the soft tissue environment. The role of gingival fibroblasts in modulating the osteogenic potential of mandible mesenchymal stem cells (MMSCs) mediated by bone substitute materials (BSMs) is not fully understood. This study aimed to investigate the impact of gingival fibroblasts on the osteogenic differentiation of MSCs in the presence of BSMs and to elucidate the underlying mechanisms, focusing on the WNT/β-catenin signaling pathway.

**Methods:**

Gingival fibroblasts and BSMs co-culture conditioned medium was used to culture MMSCs, and the expression and activity of alkaline phosphatase (ALP), as well as osteogenic and fibrogenic gene and protein expression, were evaluated. Additionally, the expression of key factors of WNT/β-catenin signaling pathway were investigated. *In vivo* animal experiments were conducted to assess the effect of gingival fibroblasts on BSM-mediated bone regeneration.

**Results:**

Gingival fibroblasts and BSMs co-culture environment did not affect MMSCs proliferation but significantly inhibited ALP expression and activity, as well as osteogenic gene and protein expression, while promoting expression of fibrogenic markers. This suppression was associated with the downregulation of key factors in the WNT/β-catenin signaling pathway. *In vivo*, increased suppression of bone defect repair was observed with higher amounts of gingival fibroblasts, confirming the *in vitro* findings.

**Conclusion:**

Our study demonstrates that gingival fibroblasts can suppress the osteogenic potential of BSMs by inhibiting the autocrine WNT expression and the activation of the WNT/β-catenin signaling pathway in MMSCs. These findings highlight the importance of considering the cellular microenvironment in tissue engineering and regenerative medicine and suggest potential targets for modulating MMSCs behavior to enhance bone regeneration.

## 1 Introduction

In the field of oral and maxillofacial surgery, the regeneration of bone tissue is a critical challenge, particularly in cases involving periodontal defects, alveolar ridge augmentation, and dental implant placement. The success of these procedures often hinges on the ability to effectively promote osteogenesis while simultaneously managing the soft tissue environment (Tan et al., 2021; Wu et al., 2019). Among the cellular components of the periodontium, gingival fibroblasts have emerged as key players in this complex biological interplay (Parisi et al., 2024). The present study is designed to explore the impact of gingival fibroblasts on the osteogenesis process mediated by bone substitute materials (BSMs) and to elucidate the underlying mechanisms.

BSMs are widely used to facilitate bone regeneration in various clinical scenarios where there is a loss of bone structure due to disease, trauma, or surgical intervention. These materials act as osteoconductive scaffolds, providing a surface for bone cell attachment, proliferation, and differentiation. However, the effectiveness of BSMs is not solely dependent on their physical properties but is also significantly influenced by the cellular microenvironment (Chen et al., 2018).

Gingival fibroblasts are the predominant cell type in the gingival stroma and contribute to the formation and maintenance of the periodontal ligament, as well as the overall integrity of the gingival tissue (Wielento et al., 2023). These cells are known to secrete a variety of cytokines, growth factors, and extracellular matrix components that can influence the behavior of other cell types, including osteoblasts and osteoclasts (Parisi et al., 2024; Wielento et al., 2023; Fadl and Leask, 2023). Recent evidence suggests that gingival fibroblasts may exert a suppressive effect on osteogenesis mediated by BSMs, potentially through the modulation of signaling pathways critical to bone formation (Ghuman et al., 2019).

The WNT/β-catenin signaling pathway is a key regulator of osteoblast differentiation and bone formation. Aberrant activation or inhibition of this pathway can lead to imbalances in bone remodeling, resulting in either excessive bone formation or osteopenic conditions (Liu et al., 2022). In the context of BSM-mediated osteogenesis, the interaction between gingival fibroblasts and the WNT/β-catenin pathway becomes particularly relevant. Our hypothesis is that gingival fibroblasts may release soluble factors or engage in cell-cell contact interactions that interfere with the WNT/β-catenin signaling in osteoprogenitor cells, thereby suppressing their differentiation into mature osteoblasts.

To address this hypothesis, the current study will investigate the direct and indirect effects of gingival fibroblasts on osteogenesis using both *in vitro* and *in vivo* models. We will assess the expression of key osteogenic markers, the secretion profile of soluble factors known to modulate the WNT/β-catenin pathway, and the functional response of osteoprogenitor cells co-cultured with gingival fibroblasts in the presence of BSMs. Additionally, we will explore the potential of targeted interventions to abrogate the suppressive effects of gingival fibroblasts, with the aim of enhancing the osteogenic potential of BSMs.

Understanding the mechanisms by which gingival fibroblasts influence BSM-mediated osteogenesis is essential for the development of novel therapeutic strategies to improve bone regeneration outcomes. By identifying the specific factors and pathways involved in this process, we can potentially manipulate the gingival microenvironment to foster a more conducive setting for bone formation. This research could lead to advancements in the field of regenerative dentistry and contribute to the improvement of clinical procedures for bone tissue repair and reconstruction.

It is important to note that while previous research has explored the role of gingival fibroblasts in osteogenesis, there are still significant gaps in our knowledge. For instance, Iwata et al. demonstrated that histone deacetylase 1 and two can regulate osteogenesis in bone marrow-derived mesenchymal stem cells co-cultured with human gingival fibroblasts and periodontal ligament cells (Iwata et al., 2023). Furthermore, Kaneda-Ikeda et al. found that human gingival fibroblasts can regulate osteogenesis via miR-101-3p in mesenchymal stem cells (Kaneda-Ikeda et al., 2020). However, these studies primarily focused on specific molecular mechanisms or interactions between particular cell types, and a comprehensive understanding of the interplay between gingival fibroblasts and the WNT/β-catenin signaling pathway in BSM-mediated osteogenesis remains limited. The present study aims to fill this gap by systematically investigating the comprehensive impact of gingival fibroblasts on MMSCs osteogenic differentiation *in vitro* and *in vivo* models, as well as their modulation of the WNT/β-catenin signaling pathway, providing deeper insights into this field.

## 2 Materials and methods

### 2.1 Preparation of bone substitute materials

Porcine hydroxyapatite (PHA) were used as bone sustitute materials (BSA) in this study. The preparation method PHA was performed according to our previous studies (Liu et al., 2018). Briefly, porcine cancellous bone harvested from the femoral epiphysis was boiled to remove macroscopic impurities. Then, the sample was dissected into regular blocks and calcinated at 800°C for 2 h in a muffle furnace (SGM6812BK, Sigma Furnace Industry, China). The thermally treated PHA blocks with original porous structure (5 mm in diameter) were used as BSA scaffold for *in vivo* test. Then, the blocks were ground into powders and 50 mg of powders were compressed into a BSA disk (diameter: 8 mm; thickness: 2 mm) using a rotary tableting machine (ZP10A, TianQi Pharmaceutical Machinery Co., China) for *in vitro* test. All samples were used after autoclaved sterilization.

### 2.2 Isolation and culture of rat GFs and rat MMSCs

Primary cells isolation were carried out in strict accordance with the Institutional Animal Care and Use Committee (IACUC) of Sun Yat-sen University and were approved by the Animal Ethical and Welfare Committee of Sun Yat-sen University (SYSU-IACUC-2018-000283). Four-week-old male Sprague Dawley (SD) rats were sacrificed by cervical dislocation for isolation of primary rat gingiva fibroblasts (GFs) and mandible mesenchymal stem cells (MMSCs).

GFs were obtained from gingival biopsies of rats. The collected tissues were rinsed with phosphate buffered saline (PBS; GIBCO, United States) supplemented with 1% penicillin-streptomycin (PS; HyClone, United States) immediately after separation from gingiva and then cut into small pieces with sterile scissors. After being dampened with Dulbecco’s modified Eagle medium (DMEM; GIBCO), tissue fragments were evenly spread out on the dish. Then, the petri dish was inverted in an incubator for overnight. Finally, the dish was flipped upright and filled with complete medium containing DMEM, 10% fetal bovine serum (FBS; GBICO, United States), 1% PS at 37°C in a 5% CO_2_ humidified atmosphere. It required about 10 days for the primary cells to reach confluence, and then, the cells were washed with PBS and passaged with trypsin (GBICO, United States). Cells from passages 2-6 were used for the experiments.

As for MMSCs, bone marrow was collected by flushing mandible with complete medium, followed by centrifugation at 1,000 rpm for 5 min at room temperature. Then, the cells were resuspended in complete medium and seeded in culture flasks. The cells were cultured at 37°C in a 5% CO_2_ humidified atmosphere. After 24 h, non-adherent cells were removed by washing with PBS twice. The adherent cells (passage 0) were cultured, and the medium was changed every 3 days. When the cells reached 80% confluence, they were passaged with trypsin (Gibco). Only early passage cells 3–5 were used in this study.

### 2.3 Preparation of co-culture conditioned medium

**FIGURE 1:**
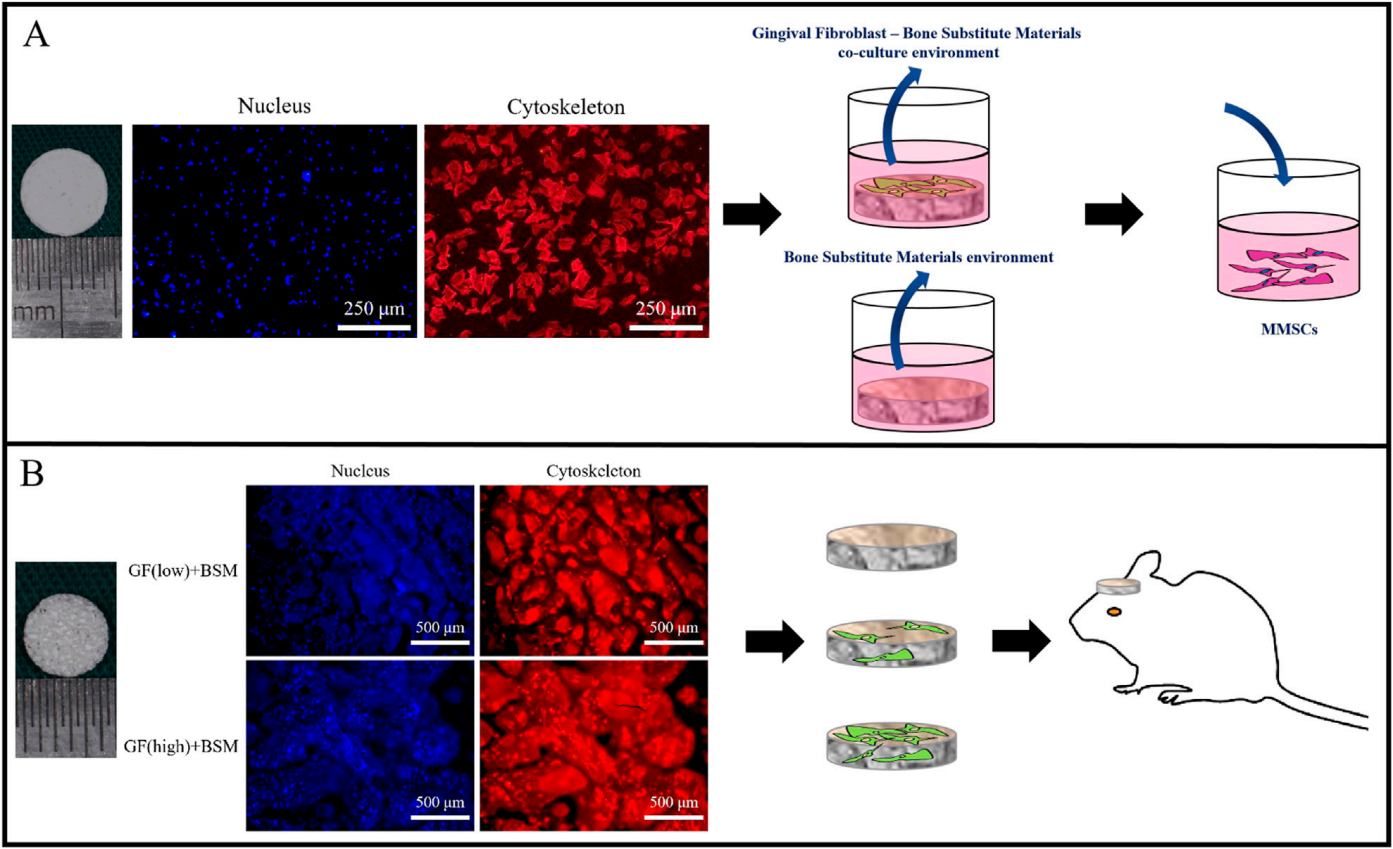
**(A)** The surface of 8 mm PHA disks to generate co-culture environment (co-colture conditoned medium). The extract medium of PHA disks served as control (conditioned medium). Both medium were used to culture MMSCs. Scale bar = 250 µm. **(B)** Different concentration of gingival fibroblasts were embedded in 5 mm PHA scaffold with original porous structure. PHA scaffold without cells served as control. All of them were implanted into 5 mm rat calvarial bone defect. Scale bar = 500 µm..

PHA disks were put in a 48-well cell culture plate and pre-immersed in DMEM for 24 h before cell seeding. The cells at a density of 5 × 10^4^/well in complete medium were seeded onto the wells containing a PHA disk. After 24 h, the medium was changed to 1 mL DMEM with 1% PS. The supernatant was centrifuged and sterilized by filtration through 0.2 μm filter membranes (Millipore, United States) after incubation for another 24 h. The harvested supernatant was supplemented with DMEM with 1% PS at a ratio of 1:2 to obtain the co-culture conditioned medium. The conditioned group were prepared following the same steps as above, except that no cell was seeded on the disk (Figure 1A).

### 2.4 Cell proliferation assay

MMSCs were seeded at a density of 2,000 cells/well in 96-well cell culture plates. After 24 h, the medium was replaced by 200 µL of co-culture conditioned medium, conditioned medium or DMEM with 1% PS. The cells were cultured with the above media for 1, 3, and 5 days. The density of cells was determined with the Cell Counting Kit-8 (CCK-8; Dojindo, Japan) according to the manufacturer’s instruction. The colorimetric change at 450 nm was analyzed using a spectrophotometric microplate reader (GENios, Germany). The results are expressed as the optical density (OD).

### 2.5 ALP staining and ALP activity detection

ALP staining and activity detection of MMSCs were assayed at 3 and 7 days after seeding in 24-well cell culture plates with 500 µL of co-culture conditioned medium or conditioned medium. Following fixation, cells were stained with BCIP/NBT alkaline phosphatase color development kit (Beyotime, China) according to the manufacturer’s instruction.

As for ALP activity detection, the cells were lysed with 0.1 M Triton X-100 (Sigma, United States) for 0.5 h and centrifuged at 10630 g for 5 min. The supernatant was collected to detect ALP activity using p-nitrophenyl phosphate as a substrate, according to the instructions for the ALP assay kit (Nanjing Jiancheng, China). The amount of p-nitrophenol product was measured spectrophotometrically at 520 nm. The protein concentration in the cell lysates was measured using the BCA Protein Assay Kit (Beyotime, China), and 1 unit of ALP activity (U/g of protein) was defined as the amount required to release 1 nM p-nitrophenol per minute per microgram of total cellular protein.

### 2.6 RT-qPCR

MMSCs were cultured in 6-well cell culture plates with 2 mL of co-culture conditioned medium or conditioned medium for 1 and/or 3 days. RT-qPCR was performed to measure the gene expression of ALP, OCN, RUNX2, BMP2, COL1A1, TGF-β1, TGF-β3, S100A4, WNT10b, LRP5 and β-catenin. Total RNA was isolated using TRIzol Reagent (Life Technologies, United States), and cDNA was synthesized with the TaKaRa PrimeScript™ Master Mix (Perfect Real Time Kit; Takara, Japan) according to the manufacturer’s instructions. Real-time RT-PCR was performed using SYBR Premix Ex TaqTM (Takara). The housekeeping gene GAPDH was used as an internal control. The primer sequences for the target genes are listed in Table 1. All reactions were run in triplicate in three independent experiments. The ΔΔCt method was used to analyze the PCR results.

**TABLE 1:** Primers used in this Study.

Target gene	Forward primer sequence	Reverse primer sequence
ALP	ATG​TCT​GGA​ACC​GCA​CTG​AAC	AGC​CTT​TGG​GAT​TCT​TTG​TCA​G
OCN	ACC​CTC​TCT​CTG​CTC​ACT​CTG​C	TAT​TCA​CCA​CCT​TAC​TGC​CCT​CC
Runx2	CAG​TAT​GAG​AGT​AGG​TGT​CCC​GC	AAG​AGG​GGT​AAG​ACT​GGT​CAT​AGG
BMP2	ATG​GGT​TTG​TGG​TGG​AAG​TG	AGT​TCA​GGT​GAT​CAG​CCA​GG
COL1A1	GGA​TCG​ACC​CTA​ACC​AAG​GC	GAT​CGG​AAC​CTT​CGC​TTC​CA
TGF-β1	CAA​TGG​GAT​CAG​TCC​CAA​AC	GTT​GGT​ATC​CAG​GGC​TCT​CC
TGF-β3	AGC​ACA​CAG​TCC​GCT​ACT​TC	TGT​GTG​AAC​CCA​GGA​ACG​AG
S100A4	CTC​TGT​TCA​GCA​CTT​CCT​CTC	TGA​GCT​CTG​TCT​TGT​TCA​GC
WNT10b	CAG​GCT​TTG​TGT​GGA​GTC​ATT	GAG​GTT​CTG​GGC​TGT​AGT​GG
LRP5	GAC​TAA​CAA​CAA​TGA​CGT​GG	ATA​GTC​TTG​AGG​CTG​ACA​TC
β-catenin	TCC​GCA​TGG​AGG​AGA​TAG​TTG	CCG​AAA​GCC​GTT​TCT​TGT​AGT
GAPDH	TTC​CTA​CCC​CCA​ATG​TAT​CCG	CAT​GAG​GTC​CAC​CAC​CCT​GTT

### 2.7 Western blot

MMSCs were plated at a density of 1.5 × 10^5^ cells per well in separate 6-well plates. After reaching around 80% confluence, the culture medium was removed and replaced by co-culture conditioned medium or conditioned medium. After 3 and/or 7 days of culture, total protein was extracted from MMSCs and analyzed by Western blot. Primary antibodies were used in this study against ALP (#ab65834, 1:5,000, Abcam, Cambridge, United Kingdom), OPN (#ab166709, 1:1,000, Abcam, Cambridge, United Kingdom), RUNX2 (#8486, 1:1,000, CST, Danvers, Massachusetts, United States), VEGF (#ab46154, 1:1,000, Abcam, Cambridge, United Kingdom), Tubulin (#EM31012-02, 1:5,000, Emarbio, Beijing, China), DKK-1 (#ab109416, 1:1,000, Abcam, Cambridge, United Kingdom) and β-catenin (#9582s, 1:1,000, CST, Danvers, Massachusetts, United States). Secondary antibodies against rabbit and mouse primary antibodies (#A0208 and #A0216, 1:1,000, Beyotime, Shanghai, China) were also used. The antibodies were diluted using a universal blocking and dilution buffer for primary and secondary antibodies (Beyotime, Shanghai, China). The protein bands were visualized using the Odyssey infrared imaging system (LI-COR Biotechnology, Lincoln, Ne, United States).The relative intensity of protein bands was analyzed by ImageJ software (National Institutes of Health, Bethesda, Maryland, United States).

### 2.8 Surgical procedure

The animal experiments were carried out in strict accordance with the Institutional Animal Care and Use Committee (IACUC) of Sun Yat-sen University and was approved by the Animal Ethical and Welfare Committee of Sun Yat-sen University (IACUC-DB-16-0103). SD rats weighing between 250 and 300 g were used in this study. Two bilateral calvarial bone defects were created with a 5-mm diameter trephine bur, grafts were inserted into the bilateral defects. The design of the *in vivo* study included 4 groups (n = 4 per group): (1) Blank group without any graft; (2) BSM group without cells; (3) GF (low)+BSM group with 5 × 10^3^ GFs; (4) GF (high)+BSM group with 5 × 10^4^ GFs. Four weeks after surgery, all animals survived the procedure without any signs of diseases or complications. The grafts and surrounding tissue were dissected. Harvested specimens were fixed with 4% paraformaldehyde in 0.1 M phosphate (pH 7.2) for 24 h (Figure 1B).

### 2.9 Histology analysis

Paraffin-embedded decalcified bone sections were stained with HE and Safranin O staining and processed for immunofluorescence (IF) of WNT10b (#bs-3662R, 1:200, Bioss, Beijing, China) and β-catenin (#sc-393501, 1:200, Santa Cruz, Dallas, United States). To perform HE staining, nuclei were first stained with Mayer’s haematoxylin (Sigma-Aldrich, St. Louis, MO, United States), followed by cell plasma and extracellular matrix staining with eosin (Sigma-Aldrich). Safranine O and Fast Green Staining Kit was used to detect new bone formation (Beyotime Biotechnology, Shanghai, China), cell nuclei were stained by weigert hematoxylin for 4 min. The weigert hematoxylin was then removed, and the sections were differentiated in acid ethanol for 15 s. After washing in running water, sections were stained with fast green for 5 min. Subsequently, sections were stained with Safranin O for 2 min. After that, the residual fast green was removed using 1% acetic acid solution, and dehydration was performed using 95% ethanol and 100% ethanol. Three randomly selected sections from the longitudinal series of each sample were analyzed. The percentage of new bone formation in the region of interest was calculated by using a computer-based image analysis system (Image-Pro Plus 6.0; Media Cybernetic). To perform IF, slides were treated with 3% H_2_O_2_ for the elimination of endogenous peroxidase activity and antigen retrieval buffers (Genetech, Shanghai, China). After blocking with normal bovine serum, the slides were incubated with WNT10b and β-catenin antibodies at 4°C overnight. Then the slides were incubated with FITC conjugated secondary antibody (Beyotime, Shanghai, China) and counterstained with Dapi (Beyotime, Shanghai, China). The sections were visualized and photographed by using an Axioskop40 microscope (ZEISS).

### 2.10 Statistic analysis

The data was expressed as the mean ± standard deviation (SD). T-test was used for statistical analysis of two-sample comparison, while one-way analysis of variance (ANOVA) followed by a Tukey’s multiple comparison post hoc test was used for the statistical analysis of multiple comparisons. All statistical analysis was performed using GraphPad Prism 6.0 software (La Jolla, CA, United States). (*, P values < 0.05 was considered significant).

## 3 Results

### 3.1 Gingival fibroblasts grew on the BSM

The GFs grew well on the BSM disks and BSM scaffolds in the complete medium. Cell immunofluorescence result showed that, 24 h after seeding, GFs stretched well and distributed equally on the BSM disks and BSM scaffolds (Figure 1).

### 3.2 The effect of microenvironment constructed with GFs and BSM on MMSCs *in vitro*

**FIGURE 2:**
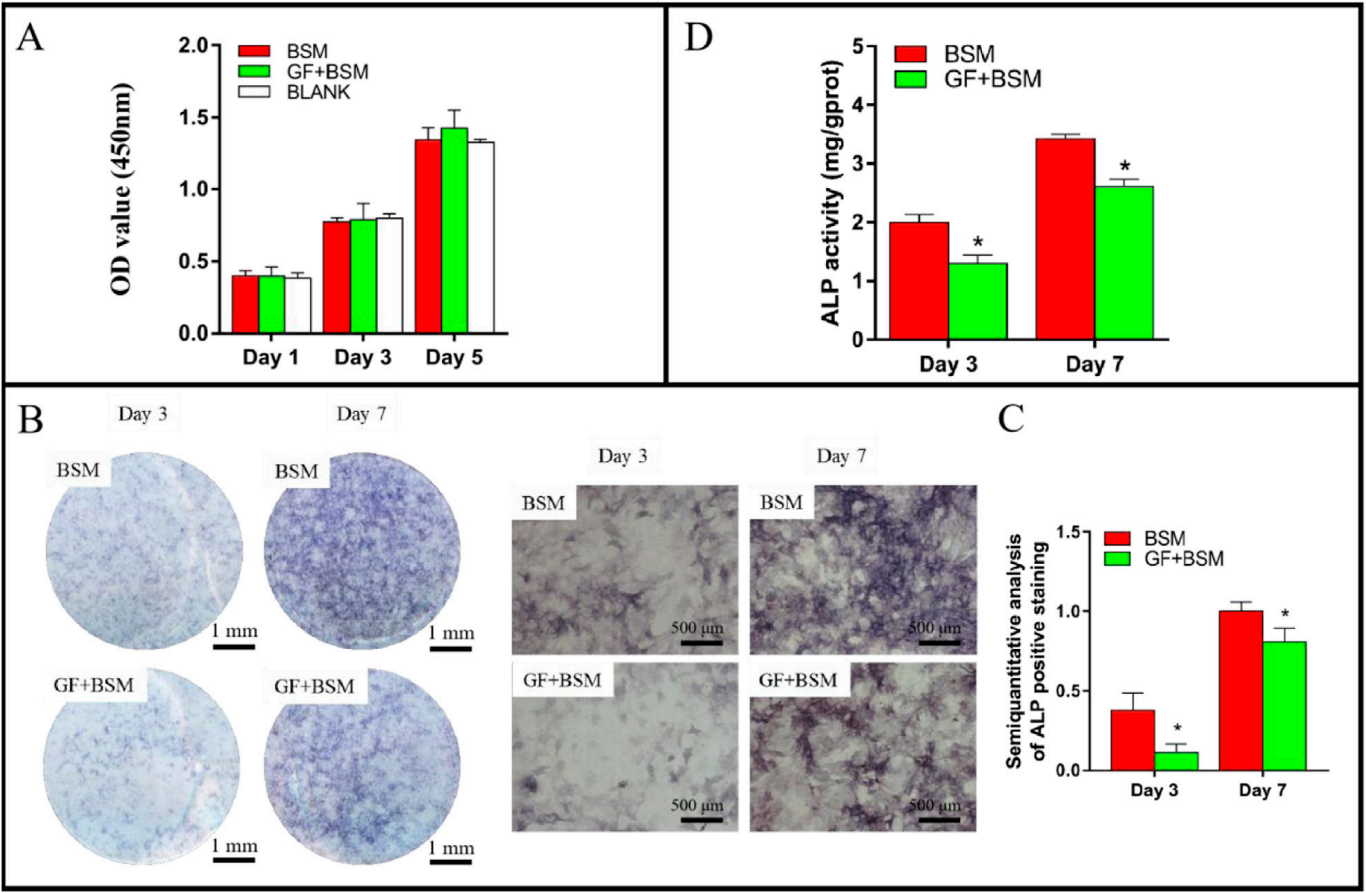
**(A)** The result of CCK-8 showed that both BSM group and GF + BSM group would not influence the proliferation of MMSCs. **(B)** ALP staining result showed that gingival fibroblast suppressed the ALP production of MMSCs. Scale bar (left) = 1 mm; Scale bar (right) = 500 µm. **(C)** Semi-quantitative analysis of ALP staining result showed that ALP production of MMSCs cultured with GF + BSM co-culture conditioned medium were significantly lower than that cultured with BSM conditioned medium. **(D)** ALP activity result showed that GF + BSM co-culture conditioned medium significantly suppressed the ALP activity of MMSCs compared to BSM conditioned medium. *Significant difference (P < 0.05) compared with BSM group.

The CCK-8 result showed that the microenvironment constructed with GFs and BSM would not influence the proliferation of MMSCs at 1, 3 and 5 days, compared to BSM group. In addition, the extracts of BSM also did not disturb the proliferation of MMSCs when compared to blank group (Figure 2A).

Both ALP staining and activity results consistently demonstrated that, the microenvironment constructed with GFs and BSM would not only significantly suppressed the production of ALP, but also remarkably weaken the activity of ALP at 3 and 7 days (Figures 2B–D).

**FIGURE 3:**
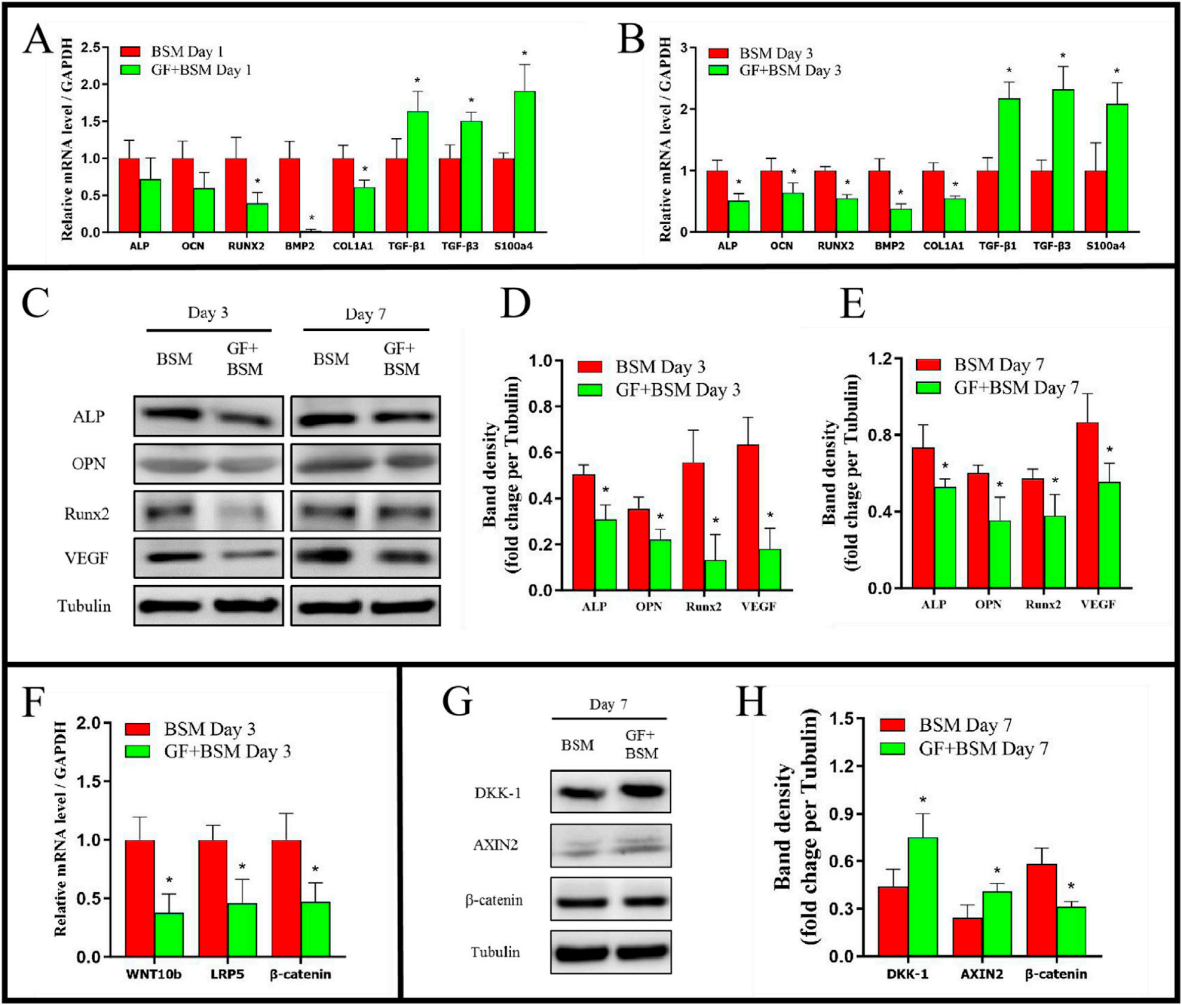
**(A, B)** The qRT-PCR results showed that GF + BSM co-culture conditioned medium downregulated the expression level of osteogenic related genes (ALP, OCN, RUNX2, BMP2 and COL1A1) and up-regulated the expression level of fibrogenic related genes (TGF-β1, TGF-β3 and S100A4) of MMSCs in day 1 **(A)** and day 3 **(B)**. **(C–E)** Western blot results showed that GF + BSM co-culture conditioned medium significantly suppressed the expression of osteogenic related proteins (ALP, OPN, RUNX2 and VEGF) in day 3 **(D)** and day 7 **(E)**. **(F–H)** The effect of GF + BSM co-culture conditioned medium on regulatory factor of WNT/β-catenin pathway. *Significant difference (P < 0.05) compared with BSM group.

qRT-PCR results showed that, at day 1, the genes expression of RUNX2, BMP2, COL1A1 of GF + BSM group were significantly lower than that of BSM group. In addition, although there were no significant differences, the expression of ALP and OCN of GF + BSM group were also lower than that of BSM group. At day 3, all the osteogenic related genes were downregulated in GF + BSM group compared to that in BSM group (Figures 3A, B). The result of Western blot showed that osteogenic proteins including ALP, OPN, RUNX2 and VEGF, which was consistent to qRT-PCR, were significantly downregulated in GF + BSM group compared to BSM group (Figures 3C–E). Interestingly, the expression of fibrogenic related genes including TGF-β1, TGF-β3 and S100A4 were remarkably upregulated in GF + BSM at day 1 and 3, which was controversial against the expression of osteogenic related genes (Figures 3A, B).

The signaling pathway analysis *in vitro* showed WNT/β-catenin pathway positive regulatory factors (WNT10b and LRP5) were significantly decreased, while the pathway negative regulatory factors (DKK-1 and AXIN2) were significantly increased, which might result in the decrease of β-catenin (Figures 3F–H).

### 3.3 The effect of microenvironment constructed with GFs and BSM on bone regeneration *in vivo*

**FIGURE 4:**
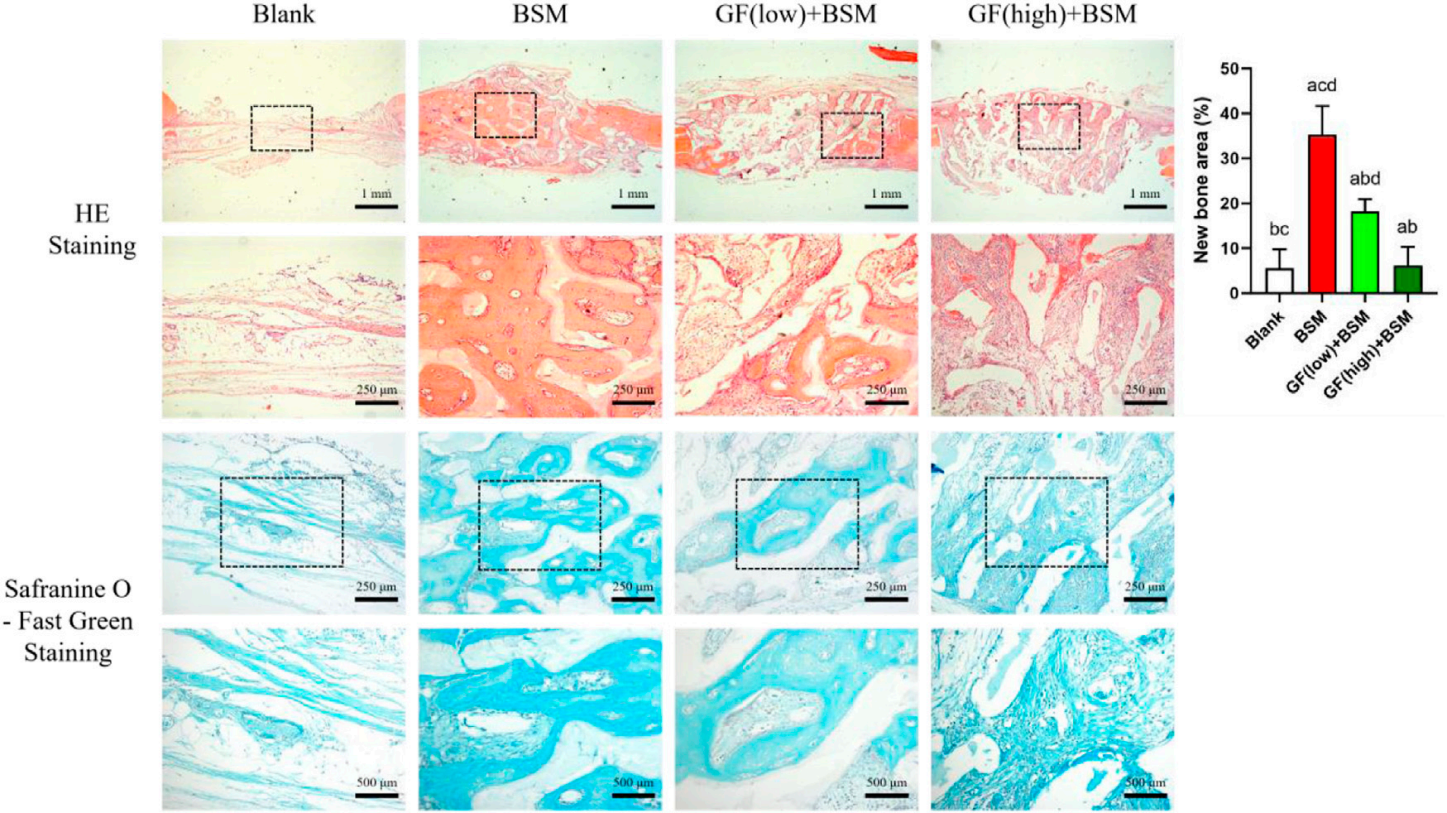
HE staining and Safranine O-Fast Green staining results showed that no new bone formed in blank group 4 weeks after the surgery, while prominently new bone tissue could be observed in BSM group. In GF (low)-BSM group, most space of defect were occupied with fibrous connective tissue, and a little new bone could be observed in the margin of the bone defect. Moreover, all space of defect were occupied with fibrous connective tissue in GF (high)-BSM group and no bone tissue could be observed. Significant differences between two groups with P < 0.05 are presented as follows: versus Blank, versus BSM, versus GF (low)+BSM, and versus GF (high)+BSM. Scale bar = 1 mm/250 µm for low magnification/high magnification in HE staining. Scale bar = 250 µm/500 µm for low magnification/high magnification in Safranine O-Fast Green staining.

HE staining result showed that, 4 weeks after the surgery, almost no new bone could be observed in blank group. In contrast, abundant newly formed bone could be found in BSM group. Interestingly, newly formed bone in BSM group was obviously more than that in both GF (low)+BSM and GF (high)+BSM groups. In GF (low)+BSM group, only a little new bone present in the margin of bone defect and the rest space of the defect was full of fibrous connective tissue. However, in GF (high)+BSM group, hardly any typical bone-like tissue could be found in the defect which was occupied with fibrous connective tissue and residual materials (Figure 4).

Safranine O-Fast Green staining was applied to further visualize the fibrous connective tissue which is stained in greyish-green. The result showed that fibrous connective tissue surrounding the material in both GF (low)+BSM and GF (high)+BSM groups were obviously more than that in BSM group. In addition, the component of cells in GF (high)+BSM group was more than that in GF (low)+BSM, which demonstrated more primitive and immature fibrous connective tissue existed in the groups with more gingival fibroblasts. New regenerated bone tissue formed and surrounded the implanted materials. Semi-quantitative analysis of new regenerated bone tissue also revealed significantly more new bone in the BSM group (35.28% ± 6.32%) than in the GF (low)+BSM group (18.17% ± 2.77%) and GF (high)+BSM group (6.17% ± 4.14%).

**FIGURE 5:**
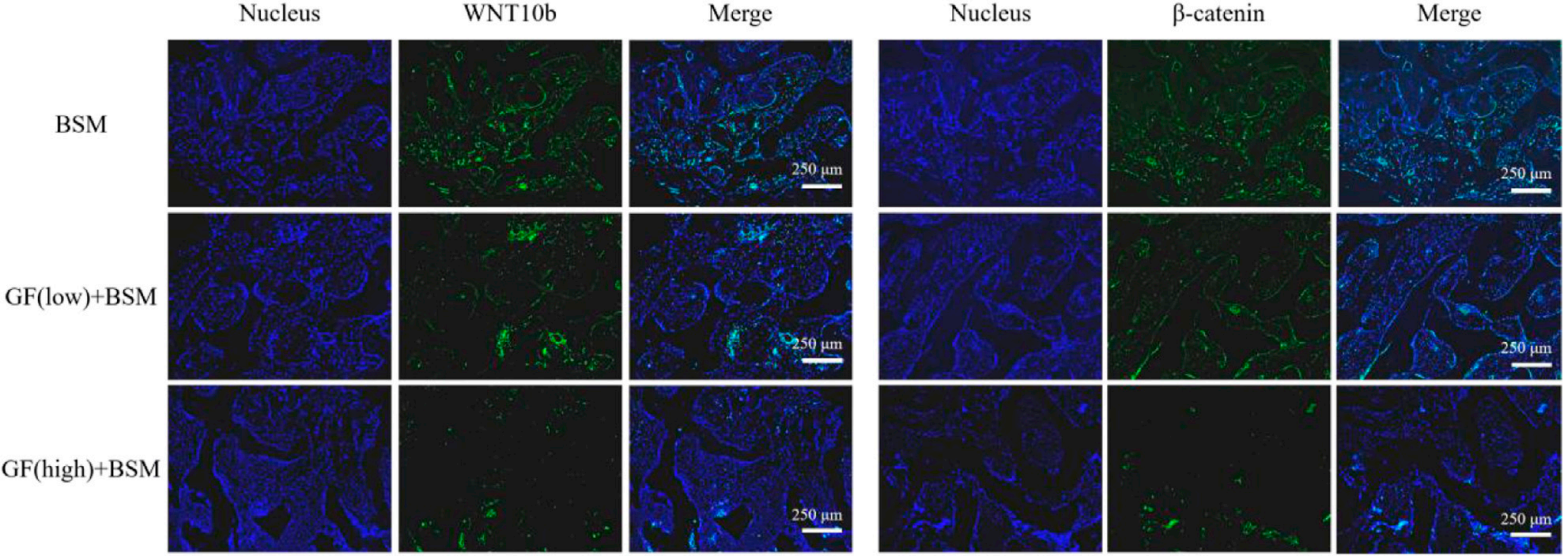
Immunofluorescence result showed that the expression level of WNT10b and β-catenin ranked according to the order: Control group (BSM) > GF (low)-BSM group > GF (high)-BSM group (scale bar = 250 μm).

The signaling pathway analysis *in vivo* with IF showed that the expression level of WNT10b and β-catenin ranked according to the order: Control group > GF (low)-BSM group > GF (high)-BSM group. This result demonstrated that the WNT/β-catenin pathway was obstructed in the presence of gingival fibroblasts and this suppression was related to the amount of gingival fibroblasts existing (Figure 5).

## 4 Discussion

In this study, we have delineated the suppressive role of gingival fibroblasts on the osteogenesis process mediated by bone substitute materials (BSMs), a phenomenon observed in both *in vitro* and *in vivo* experimental models. Our findings provide valuable insights into the complex interplay between gingival fibroblasts and the hard tissue regeneration process, which is crucial for the development of more effective bone regeneration strategies.

The *in vitro* cell experiments were designed to mimic the interaction between gingival fibroblasts and BSMs, followed by the assessment of the impact of the gingival fibroblasts and BSMs co-culture environment on the osteogenic differentiation of mesenchymal stem cells (MSCs). The observation that both BSMs environment and the gingival fibroblasts and BSMs co-culture environment did not affect the proliferation of MSCs suggests that the suppressive effect is not due to a general cytotoxicity but rather to a specific inhibition of osteogenic differentiation pathways. This is further supported by the finding that the gingival fibroblasts and BSMs co-culture conditioned medium significantly inhibited the expression and activity of alkaline phosphatase (ALP), a key enzyme in the osteogenic differentiation process. Our findings are consistent with the current understanding in the field, which suggests that gingival fibroblasts have a high regenerative capacity and are involved in various biological processes, including tissue repair and immune modulation. These cells are known to produce extracellular matrix components and play a critical role in promoting tissue integrity and repair instead of affecting the growth and proliferation functions of other cells (Fadl and Leask, 2023).

Our data reveal gingival fibroblasts and BSMs co-culture conditioned medium’s dualistic effect on MMSC differentiation, with a suppression of osteogenic genes and proteins such as ALP, OCN, RUNX2, BMP2, COL1A1 and VEGF while promoting the expression of fibrogenic markers such as TGF-β1, TGF-β3 and S100a4. This shift towards a fibrogenic phenotype suggests that gingival fibroblasts may alter the environment in a way that favors soft tissue formation over bone regeneration. The preferential activation of fibrogenic pathways could be a response to the presence of BSMs, which may be perceived as a foreign entity that triggers a wound healing-like response rather than a bone-forming signal.

While our study focuses on the overall impact of gingival fibroblasts on osteogenesis, it is worth speculating about the specific factors they produce that could inhibit bone formation. Potential candidates include cytokines such as TGF-β1 and TGF-β3, which are known to promote fibrogenesis and may compete with osteogenic signals (Ong et al., 2021) Additionally, the secretion of matrix metalloproteinases (MMPs) by gingival fibroblasts could degrade the extracellular matrix, thereby disrupting the osteogenic niche (Checchi et al., 2020). Other factors, such as S100A4, which is upregulated in our co-culture model, may also play a role in suppressing osteogenesis (Wen et al., 2022). Future research should aim to identify and characterize these factors, as well as explore their mechanisms of action. This will not only enhance our understanding of the complex cellular interactions in bone regeneration but also provide new targets for therapeutic interventions.

Some studies have found the presence of gingival mesenchymal stem cells (GMSCs) in gingival soft tissue. GMSCs have emerged as a promising cell source for tissue engineering and regenerative medicine due to their unique biological properties. GMSCs exhibit high proliferation rates, low immunogenicity, and multilineage differentiation potential (into osteoblasts, adipocytes, and chondrocytes), and they have been successfully used in animal models for bone regeneration (Dave et al., 2022; Kim et al., 2021; Peng et al., 2024). In clinical practice, we have observed that if the barrier function of the membrane utilized in guided bone regeneration is not maintained effectively over a sufficient duration, the predominant fibroblasts may hinder the bone regeneration process. Therefore, the presence of a large number of gingival fibroblasts in the gingival tissue can significantly influence the behavior of GMSCs, potentially limiting their osteogenic differentiation (Wen et al., 2022; Dave et al., 2022; Kim et al., 2021). This highlights the need for strategies to either enhance the osteogenic potential of GMSCs or to modulate the local cellular environment to favor bone formation over fibrous tissue development.

While our study focuses on the crosstalk from gingival fibroblasts to MSCs, it is important to note that crosstalk can be a two-way street. As demonstrated by Zhao et al., the conditioned medium from MSCs can also influence the behavior of human gingival fibroblasts, affecting their proliferation and collagen synthesis (Zhao et al., 2017). This two-way interaction highlights the complexity of the cellular microenvironment and the need to consider the reciprocal influences between different cell types in tissue engineering and regenerative medicine. Future studies should explore the bidirectional interactions between gingival fibroblasts and MSCs to fully understand their roles in bone regeneration.

The downregulation of key factors in the WNT/β-catenin signaling pathway in MSCs treated with the gingival fibroblasts and BSMs co-culture conditioned medium is a critical finding of our study. The WNT/β-catenin pathway is a well-established regulator of osteogenic differentiation, and its inhibition could explain the observed suppression of ALP expression and activity (Hu et al., 2024; Kubota et al., 2010). This pathway is also implicated in the maintenance of MSC lineage commitment, making it a likely target for the crosstalk between gingival fibroblasts and MSCs. The inhibition of this pathway could lead to a default towards a fibrogenic lineage, which is characterized by a lower osteogenic potential (Chen et al., 2016; Soundararajan and Kannan, 2018; Lee et al., 2006). MSCs are known to secrete WNT proteins as part of an autocrine signaling mechanism that activates the WNT/β-catenin pathway and promotes osteogenesis (Liu et al., 2022; Mankuzhy et al., 2023; Han et al., 2022; Ling et al., 2009). Our study detected the autocrine secretion of WNT proteins by MSCs, which are essential for activating the WNT/β-catenin pathway and promoting osteogenesis. However, in the presence of gingival fibroblasts and BSMs, the autocrine secretion of WNT-related genes and protein by MSCs was found to be inhibited.

The *in vivo* animal experiments provided compelling evidence that supports the *in vitro* observations. The increased suppression of bone defect repair with higher amounts of gingival fibroblasts suggests a dose-dependent relationship, which has practical implications for clinical applications where the ratio of gingival fibroblasts to MSCs may be a critical factor in the success of bone regeneration procedures. The immunofluorescence results from tissue sections further validated the *in vitro* observations, showing that gingival fibroblasts can indeed suppress the activation of the WNT/β-catenin signaling pathway *in vivo*. This suppression likely underlies the observed inhibition of MSC osteogenic differentiation and highlights the importance of considering the local cellular environment when designing strategies for bone regeneration.

These findings contribute to a growing body of evidence that the cellular environment plays a crucial role in tissue regeneration. The ability of gingival fibroblasts to modulate the behavior of MSCs has important implications for the development of biomaterials and regenerative therapies. For instance, it may be possible to design BSMs that can neutralize the inhibitory effects of gingival fibroblasts or even harness their influence to promote a more favorable healing response.

The inhibitory effect of gingival fibroblasts on MSC osteogenic differentiation also raises questions about the broader implications for tissue engineering and regenerative medicine. It suggests that the success of regenerative therapies may depend not only on the properties of the biomaterials used but also on the complex interactions between different cell types within the regenerative niche. This highlights the need for a more holistic approach to tissue engineering, one that takes into account the full spectrum of cellular interactions that occur during the regenerative process (Armstrong and Stevens, 2019).

It is important to note that the pathways governing osteogenesis are complex. While the present study focuses on the WNT/β-catenin pathway, it is worth acknowledging the broader complexity that merits future work. Other signaling pathways, such as BMP, TGF-β, and Notch, also play significant roles in osteogenesis and may interact with the WNT/β-catenin pathway in ways that are not yet fully understood (Chen et al., 2012; Cui et al., 2022; Zhu et al., 2024). Future research should aim to explore these interactions and their potential to modulate the osteogenic process in the presence of gingival fibroblasts and BSMs.

**FIGURE 6:**
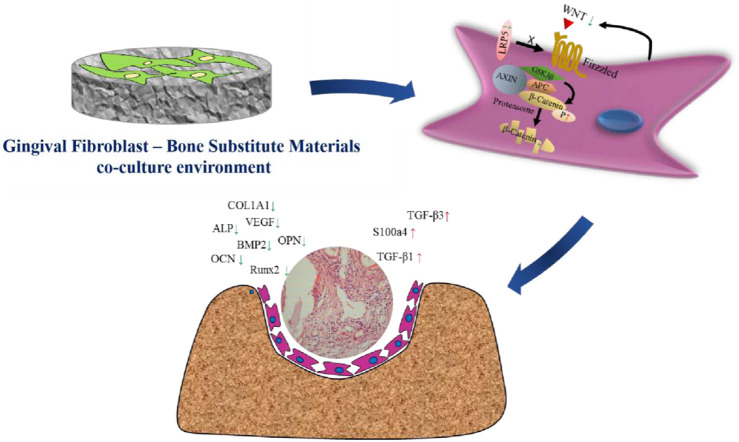
Schematic illustration of the suppress effect of gingival fibroblasts on the osteogenesis process mediated by bone substitute materials. The microenvironment constructed with gingival fibroblast and bone substitute materials suppressed the activation of WNT/β-catenin pathway, thus down-regulating the expression of osteogenic factors as well as up-regulating the expression of fibrogenic factors. Finally, the osteogenesis function mediated by bone substitute materials would be obstructed and the growth of fibrous connective tissue would be enhanced.

In conclusion, our study provides compelling evidence that gingival fibroblasts can suppress the osteogenic potential of BSMs by inhibiting the WNT/β-catenin signaling pathway in MSCs (Figure 6). These findings have important implications for the design of more effective bone regeneration strategies and underscore the importance of considering the cellular microenvironment in tissue engineering and regenerative medicine. Future research should focus on elucidating the specific molecular mechanisms underlying the interactions between gingival fibroblasts and MSCs, as well as exploring potential strategies to modulate these interactions to enhance bone regeneration. Additionally, the development of novel biomaterials that can either mimic the natural bone environment or actively promote osteogenic differentiation while suppressing fibrogenic responses could represent a significant advancement in the field. Understanding and potentially manipulating the cellular crosstalk within the regenerative niche may lead to more effective therapies for bone tissue repair and reconstruction.

## Data Availability

The original contributions presented in the study are included in the article/supplementary material, further inquiries can be directed to the corresponding author.
